# Factors predicting willingness to quit snus and cigarette use among young males

**DOI:** 10.1038/s41598-023-42233-8

**Published:** 2023-09-13

**Authors:** Maria Danielsson, Anelma Lammi, Simo Siitonen, Jukka Ollgren, Liisa Pylkkänen, Tuula Vasankari

**Affiliations:** 1 The Finnish Defence Forces, Kasarminkatu 17, 00130 Helsinki, Finland; 2 Doctoral Programme in Population Health, University of Helsinki, P.O. BOX 3, 00014 Helsinki, Finland; 3 Finnish Lung Health Association (FILHA), Filha Ry, Sibeliuksenkatu 11 A 1, 00250 Helsinki, Finland; 4 National Institute for Health and Welfare, P.O. BOX 30, 00271 Helsinki, Finland; 5 Finnish Medicines Agency Fimea, Helsinki, Finland; 6 Division of Medicine, Department of Oncology, Turku University Hospital and University of Turku, P.O. BOX 52, 20521 Turku, Finland; 7 Division of Medicine, Department of Pulmonary Diseases and Clinical Allergology, Turku University Hospital and University of Turku, P.O. BOX 52, 20521 Turku, Finland

**Keywords:** Epidemiology, Human behaviour, Motivation, Social behaviour, Epidemiology, Addiction, Health care, Medical research

## Abstract

The health hazards of smoking are well recognised and recently knowledge about the harmful effects of nicotine and snus is accumulating. We investigated the factors increasing the willingness of young Finnish males to quit snus and cigarette smoking. We conducted a questionnaire study conducted in 3 out of 16 Finnish Defence Forces units which included 6508 male conscripts, of whom 4706 responded (response rate 72%, mean age 19.4 years). Factors related to the willingness to quit use were analysed by ordinal regression models. Backward selection following the Akaike information criterion (AIC) was used for the model. The prevalence figures of daily snus use and smoking were 17% and 25%, respectively. 16% of the daily snus users were also daily smokers and 29% were occasional smokers. Multivariate analysis showed that the willingness to quit snus use was associated with the perception of health hazards (OR 3.09, 95% CI 1.94–4.93) and with ≥ 2 quit attempts (OR 3.63, 95% CI 2.44–5.40). The willingness to quit smoking was associated with ≥ 2 quit attempts (OR 3.22, 95% CI 2.32–4.49), and with advice to quit smoking (OR 1.65, 95% CI 1.17–2.32). We created a brief two-question assessment model for snus dependence. With this model, nicotine dependence of daily snus users was congruent with that of nicotine dependence of smokers. A direct comparison with serum cotinine levels is necessary before our assessment model can be used as a proxy for dependence. Regular snus use predisposes to nicotine addiction and accumulated health hazards. Our findings underscore the importance of health promotion efforts in early adolescence and of active support for quitting snus use. Easily applicable tools to estimate nicotine addiction are needed for everyday clinical use.

## Introduction

Nicotine is a highly addictive substance and the main dependence-causing factor among smokers. The development of nicotine addiction is a complex socio-physiological process affected by several factors, such as duration and quantity of exposure to nicotine, socioeconomic factors, educational factors and genetic predisposition. Late onset of tobacco consumption and higher education appear to be protective factors against regular nicotine use^1,2^.

The prevalence of smoking has decreased locally in countries that have taken active tobacco control efforts, and globally as a result of persistent and determined preventive work^3–6^. Nevertheless, only six countries in Europe are expected to reach the goal set by the WHO Global Action Plan for prevention and control of noncommunicable diseases of a 30% reduction in any tobacco use by the year 2025^7^. Alternative tobacco and nicotine products have been introduced by the tobacco industry in response to the diminishing popularity of smoking. The readiness to use snus (Swedish type of smokeless tobacco) has increased particularly among the younger population in the Northern European countries and the US^5,8–11^. The prevalence of smoking in Sweden and Norway is low but current tobacco use is high: 29.8% of males and 18.2% of women use any tobacco product at least occasionally^6^. The trend is similar in Finland: in 2021, approximately 10% of young males used snus daily and 6% smoked daily^5,12^.

The health hazards of smoking are well recognised. Previous studies show that younger age, lower dependence profile, previous quit attempts to quit and awareness of the smoking-related health hazards are all factors associated with an increased motivation to quit smoking^13,14^. Also the preferred smoking cessation methods among young adults and their effects have been described previously^15^. However, this knowledge is lacking for snus.

Several studies show that snus use increase overall morbidity and mortality, and public awareness about the harmful effects of nicotine and snus on health is increasing^16,17^. The bioavailability of snus is high, and the absorption of nicotine persists for as long as snus is kept buccally in the oral cavity^18–21^. Although there are interindividual differences that affect the risk of a person becoming addicted to nicotine, the amount and frequency of nicotine introduced into the body are key^18,22^.

Heatherton et al.^23^ developed and validated a useful two-question test—the Heaviness of Smoking Index (HSI)—for evaluating the nicotine dependence of smokers. In 1995, Boyle et al. developed two sets of questions with 9 and 10 items to test nicotine dependence of smokeless tobacco users^24^. In both studies a strong association between the levels of cotinine, a nicotine metabolite, and the use of smokeless tobacco within 30 min of waking up was observed. Boyle et al.^24^ confirmed previous observations that duration and frequency of use of smokeless tobacco correlated significantly with cotinine. Later in 2006 Ebbert et al.^25^ introduced a six-question dependence test. Currently, a modified six-question questionnaire is recommended by the Finnish Institute for Health and Welfare for the evaluation of snus dependence in Finland^26^. However, these long questionnaires are not very well applicable for daily clinical practice, and more user-friendly tools are welcomed.

This study aims to investigate the factors associated with the willingness of snus users and smokers to stop their habit. A preliminary two-question dependence test for snus users, analogous to the above-mentioned HSI^23^, is also introduced.

## Methods

The methodology has previously been described in detail by Danielsson et al. in 2019^8^ and is reviewed briefly in the three sections below.

The study was conducted among young males and females conscripts at the beginning of their military service in three out of sixteen Finnish Defence Forces units (the Guard Jaeger Regiment, the Karelian Brigade, and the Kainuu Brigade). These units train recruits coming from different parts of Finland. The troops were selected by simple random sampling.

### Sampling

The study population consisted of 6508 male conscripts chosen by simple random sampling and covering the two annual contingents (January and June) in the years 2014, 2015 and 2016. A total of 4706 (in 2014 n = 1916, in 2015 n = 1510, and in 2016 n = 1280) males answered the questionnaire, giving in a total response rate of 72%. Female respondents, n = 124 (2.6%), were excluded from the main analyses due to the low number of female conscripts. For female respondents, only some baseline characteristics were collected.

### Data collection and measurement

The conscripts received a questionnaire-based survey during their first week of service in conjunction with a general health inspection. Basic questions on age, gender and educational background and on the use of snus, cigarettes, and electronic cigarettes were included. The questions assessing the starting age of daily smoking and snus use were limited to the respondents who reported a starting age above 9 years.

The history of smoking and snus use was classified into four categories: daily, occasional, former, or never. The respondents’ educational level was categorised into three groups: comprehensive, vocational, and upper secondary education. The duration of smoking and snus use was calculated from the current age and starting age of smoking/snus use.

Earlier quit attempts were classified into three categories: none, once, and twice or more. The respondents’ opinion on the harmfulness of smoking/snus was categorised into four groups ranging from 1 “not at all harmful” to 4 “very harmful” and their opinions on addictiveness into three groups ranging from 1 “not at all addictive” to 3 “very addictive”. The willingness to quit smoking/snus use was categorised as: no intention to quit, intention to quit within 6 months, or intention to quit later than 6 months. The question on by whom the respondent had been advised to quit smoking/snus use within the past 12 months was categorised as: physician, nurse, health care professional, dentist, pharmacist, family member, or someone else.

The two-question nicotine dependency test for smokers, the HSI, was used to estimate the level of nicotine dependence among smokers. The HSI test categorises nicotine dependence into four categories: low 0–1 points, moderate 2 points, strong 3 points, and very strong 4–6 points^27^.

To estimate the level of nicotine dependence among snus users we used a two-question dependence test (Table 1). The score for the time between wake-up and placement of the first snus portion into the mouth was analogous with HSI i.e., the time to first cigarette of the day: the first snus placed intraorally in less than 6 min gave 3 points, within 6–30 min 2 points, within 31–60 min 1 point and over 60 min 0 points.

**Table 1:** Two-question nicotine dependence test for smokers and snus users.

Question	Smokers	Snus users	Score
Time (min) to first cigarette /snus portion of the day	< 6	< 6	3
	6–30	6–30	2
	31–60	31–60	1
	> 60	> 60	0
Number of cigarettes or snus portions used per day*	≤ 10	1–7	0
	11–20	8–12	1
	21–30	> 12	2
	> 30	*	3
Total score and interpretation:	0–1 = low nicotine dependence 2 = moderate dependence 3 = strong dependence 4–6 = very strong dependence		

*Only 17% used more than 12 snus portions per day. Therefore, a three-category coding for snus users based on the six-question nicotine dependence test was used^26^.

For scoring of the number of snus portions used per day three categories were used (Table 1): 1–7 portions per day 0 points, 8–12 portions 1 point and more than 13 portions 2 points. This categorisation of snus use was adapted from the corresponding question as in the six-question nicotine dependence test for snus users used in Finland^26^. To ensure a sufficient cumulative number of responses in each category, a third point concerning the number of snus portions used per day was necessary to omit from the scoring as opposed to the HSI.

Based on these two questions, *time to first snus portion of the day* and *number of snus portions used per day*, nicotine dependence was categorised as follows: low dependence 0–1 points, moderate dependence 2 points, strong dependence 3 points, and very strong dependence 4–5 points. In 2014, these two questions were not included in the first questionnaire for snus users, and therefore the first out of the two cohorts in 2014 is excluded from the dependence analyses. Sensitivity analyses showed that this did not affect the outcome.

### Statistical analyses

The linear-by-linear association test for trend was used to evaluate the association between snus use or smoking and education. Models with ordinal regression were applied to calculate the odds ratios (OR) for the willingness to quit daily snus and cigarette use (reference: no intention/willingness to quit) after 6 months and within 6 months. Univariate analyses were used for the following explanatory variables for snus users and smokers separately: age; educational level; dual-use; duration of cigarette and snus use; perception of harmfulness; attempts to quit; perception of the addictiveness of cigarettes and snus; and level of dependence. Those variables that had a p value < 0.2 were included in the preliminary multivariate model. The variables for the final multivariate model were selected by the backward selection using the Akaike information criterion (AIC)^28,29^. We used the proportional odds assumption in the ordinal regression model, and it was tested by the Brant test.

In the preliminary multivariate model where the dependence variable for snus use was included, the p value for dependence was the highest (p = 0.51) of all variables in the model. When the dependence variable was excluded, the AIC dropped clearly (by 3.7) and the Bayesian information criterion (BIC) dropped by 14.4. Thus, there was no evidence that the dependence variable for snus should be included in the final multivariate model.

Multiple imputation by chained equations (MICE) was used to study the stability of the models because of the missing data to avoid any bias and power loss of the complete case analysis^30,31^. Multiple imputation assumed that the data’s missingness was missing at random (MAR). Data were robust irrespective of the study year. The results were not age-adjusted since the age distribution of the study population was homogeneous.

All analyses were performed using the SPSS software (version 23.0, SPSS, Inc., Chicago, IL, USA), and the Stata statistical software for data science (version 16.1). A p value < 0.05 was considered statistically significant. All methods were applied in accordance with the relevant guidelines and regulations.

### Ethical approval and informed consent

The study protocol was evaluated by the medical ethics committee of the Helsinki and Uusimaa Hospital District and obtained a favourable opinion (Number 148/13/03/00/2013). The Finnish Defence Forces gave permission to perform the study. Answering the survey was voluntary. Each recruit was first provided with written and verbal information about the survey after which they were asked to sign an informed consent. All data were processed according to the EU general data protection regulation (EU2016/679).

## Results

The study cohort consisted of 4706 male and 124 female respondents whose mean age was 19.4 years (± SD1.2) and 20.2 (± SD 2.4), respectively. Among males, the overall prevalence of snus use was 35%, and the prevalence of daily use was 17%. The corresponding figures for smoking were 38% and 25% (Table 2).

**Table 2:** Snus use and smoking habits in relation to educational background among study subjects.

	Total N = 4706	Daily snus users N = 784**	Daily smokers N = 1172***
Mean age	19.4, SD ± 1.2	19.3, SD ± 0.7	19.5, SD ± 1.3
Educational level^a,b^			
Comprehensive school*	438 (9.3%)	71 (16%)	239 (55%)
Vocational school	1925 (41%)	309 (16%)	661 (34%)
Upper secondary education	2315 (49%)	349 (15%)	190 (8%)
Snus use	*Exclusive*		
Daily user	784 (17%)		**128 (11%)**
Occasional	864 (18%)		**491 (42%)**
Former	286 (6%)		112 (10%)
Never user	2726 (58%)		431 (37%)
Smoking	*Exclusive*		
Daily smoker	1172 (25%)	**128 (16%)**	
Occasional	627 (13%)	**229 (29%)**	
Former	420 (9%)	159 (20%)	
Never smoker	2455 (52%)	264 (34%)	

^a^There was no correlation between educational level and snus use (linear-by-linear association test for trend p = 0.135). ^b^There was a strong correlation between educational level and smoking (linear-by-linear association test for trend p < 0.001). Data on dual use (snus and cigarettes) is shown by bolding. Never smoker/snus user is defined as having no history of regular use. Missing *n = 20, **n = 46, ***n = 32.

The overall prevalence of snus use among females was 7% (n = 9), and the prevalence of daily use was 4% (n = 5). The corresponding figures for smoking were 24% (n = 29) and 13% (n = 6). Females were not included in further analyses since they were so few; no general conclusions on their smoking and snus habits can be drawn.

Approximately 16% of daily male snus users reported simultaneous daily smoking and 29% reported occasional smoking. Over half of the daily smokers were dual users: 11% used snus daily and 42% occasionally. Over half of the daily smokers (55%) had undergone comprehensive school education, while the snus users were equally distributed among the three different educational categories. There was no correlation between the educational level and snus use was found (p = 0.135). (Table 2).

### Perceptions of harmfulness of snus use and smoking on health

Over half of the respondents or 58% (2600/4480) acknowledged that snus is harmful to health, but only 21% (956/4480) considered snus very harmful. A third (33%, 1466/4480) perceived snus as neither harmful nor harmless, and 9% (414/4480) considered snus harmless.

Most of the smokers, 88%, regarded smoking as clearly harmful (43%, 1959/4509) or very harmful (45%, 2012/4509) to health. A tenth (11%, 476/4509) regarded smoking as neither harmful nor harmless and only 1% regarded smoking as harmless to health.

### Perceptions of addictiveness of snus use and smoking

Snus was considered to be very addictive (29%, 1279/4460) or clearly addictive (36%, 1593/4460) by approximately 65% of the respondents, while 26% (1159/4460) regarded snus as being neither addictive nor nonaddictive. Only 9% (429/4460) considered snus not to be addictive at all.

Most smokers recognised smoking as addictive. Almost 80% considered smoking to be very addictive (38%, 1715/4499) or clearly addictive (41%, 1824/4499) and only 5% (238/4499) regarded smoking as non-addictive.

### Nicotine dependence of smokers and snus users

The mean amount of snus consumption was seven portions (± SD 5.6) per day among daily snus users. Approximately 40% (308/767) used 1–7 and 43% (329/767) used 8–12 portions per day. Almost a fifth (17%, 121/767) used more than 12 snus portions per day. Daily smokers smoked, on average, 10 cigarettes (± SD 7.8) per day. Approximately 55% (635/1161) smoked 1–10 cigarettes and 39% (454/1161) smoked 11–20 cigarettes per day. Only 6% (72/1161) smoked more than 20 cigarettes per day.

**Figure 1:**
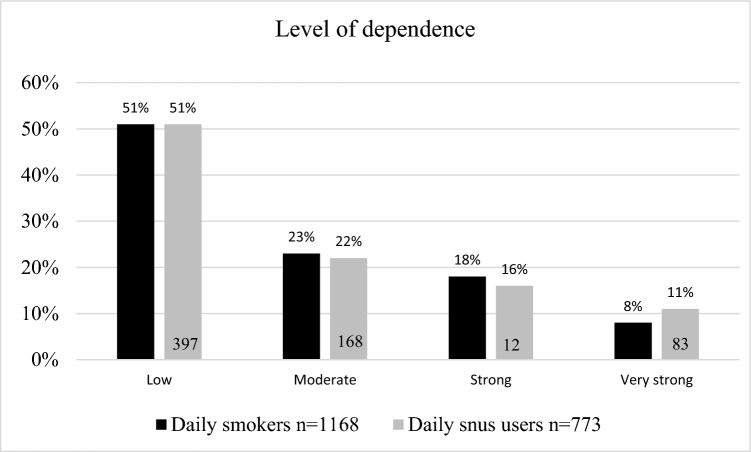
Cigarette and snus dependence among daily users. Missing values of smokers n = 4/1172 and daily snus users n = 11/784.

Half (51%, 597/1168) of the daily smokers reported low nicotine dependence, while 23% (267/1168) reported moderate dependence and 26% (304/1168) strong or very strong dependence (Fig. 1).

The profile of daily snus users closely resembled that of smokers; 51% (397/773) reported low dependence, 22% (168/773) moderate dependence, and 27% (208/773) strong or very strong dependence (Fig. 1).

### Factors predicting willingness to quit snus use among daily users

Almost half (45%, 325/729) of daily snus users expressed a willingness to quit using snus. By univariate analysis, the duration of use (OR 0.85, 95% CI 0.78–0.93) and a very strong level of dependence **(**OR 0.53, 95% CI 0.32–0.87) were inversely associated with the willingness to quit. On the other hand, perceived harmfulness of use, earlier quit attempts, and getting the advice to quit using snus were associated positively with the willingness to quit (Table 3).

**Table 3:** Factors predicting willingness to quit daily snus use (daily snus users N = 784).

Variable	Univariate analysis				Multivariate analysis		
	N^1^ (%)	OR^a^	95% Cl	p value	OR^b^	95% Cl	p value
Age (in years)							
Mean 19.3, SD ± 0.7	733	1.07	0.90–1.27	0.468			
Educational level^a^	Total	729		0.648			
Comprehensive school	71 (10%)	1					
Vocational school	309 (42%)	1.20	0.72–1.99	0.485			
Upper secondary education	349 (48%)	1.26	0.77–2.09	0.358			
Dual use of snus and cigarettes	729	0.83	0.56–1.23	0.341			
Duration of snus use (years)^b^							
Mean 3.24, SD ± 1.7	729	0.85	0.78–0.93	< 0.001	0.91	0.83–1.00	0.059
Perceived harmfulness of use							
Total	721			< 0.001			
Not at all	148 (21%)	1			1		
Neither harmless nor harmful	313 (43%)	1.80	1.19–2.72	0.005	1.72	1.09–2.72	0.021
Clearly harmful	260 (36%)	3.19	2.09–4.86	< 0.001	3.09	1.94–4.93	< 0.001
Attempts to quit use							
Total	702			< 0.001			
No attempts	379 (54%)	1			1		
One	165 (24%)	3.29	2.29–4.71	< 0.001	2.67	1.81–3.94	< 0.001
Two or more	158 (23%)	4.17	2.88–6.05	< 0.001	3.63	2.44–5.40	< 0.001
Advice to quit snus use							
Total	670	1.54	1.07–2.21	0.019	1.25	0.90–1.99	0.145
Perceived addictiveness of snus							
Total	724			0.141			
Not at all	66 (9%	1					
Neither nor	193 (27%)	1.26	0.71–2.24	0.431			
Very addictive	465 (64%)	1.57	0.92–2.67	0.096			
Level of dependence*							
Total	729			0.054			
No/low dependence	364 (50%)	1					
Moderate	162 (22%)	0.91	0.63–1.30	0.596			
Strong	122 (17%)	0.73	0.49–1.10	0.133			
Very strong	81 (11%)	0.53	0.32–0.87	0.013			

The relative order of the outcome values used in the regression: not willing to quit; willing to quit after 6 months; willing to quit within 6 months.

OR^a^ Univariate analysis: information missing for 51/784 cases.

OR^b^ Multivariate analysis: information missing for 105/628 cases.

*Excluding the first cohort of two cohorts in 2014.

Multivariate analysis showed that perceived harmfulness was associated with the willingness to quit: the OR was 3.09 (95% CI 1.94–4.93) for those who considered snus very harmful. One quit attempt within the past 6 months was associated with willingness to quit, OR 2.67 (95% CI 1.81–3.94) and two or more attempts even more so, OR 3.63 (95% CI 2.44–5.40). Among snus users, being advised to quit snus use increased the OR only by 25% (OR 1.25, 95% CI 0.90–1.99), but not statistically significantly so. The preliminary multivariate analysis showed that perceived addictiveness of snus and the level of snus dependence were not associated with the willingness to quit. Therefore (based on the AIC and BIC criteria) these two variables were excluded from the final multivariate analyses regarding the willingness of snus users to quit their habit (Table 3).

### Factors predicting willingness to stop smoking among daily smokers

Almost half (49%, 533/1090) of the daily smokers expressed a willingness to stop smoking. By univariate analysis, the level of dependence was inversely associated with the willingness to quit. Earlier quit attempts, perception of the harmfulness and addictiveness of smoking and receiving advice to quit smoking were associated with the willingness to stop smoking. (Table 4).

**Table 4:** Factors predicting the willingness to stop smoking (daily smokers N = 1172).

Variable	Univariate analysis				Multivariate analysis		
	N^a^ (%)	OR^b^	95% Cl	p value	OR^b^	95% Cl	p value
Age (in years) cigarette users							
Mean 19.5, SD ± 1.3	1 095	1.12	1.02–1.22	0.014			
Educational level^a^							
Total	1 090			0.674			
Comprehensive school (reference)	239 (22%)	1					
Vocational school	661 (61%)	1.11	0.83–1.48	0.479			
Upper secondary education	190 (17%)	1.17	0.81–1.68	0.396			
Dual use of cigarettes and snus	1 086	1.25	0.86–1.82	0.236			
Duration of smoking (years)							
Mean 4.85, SD ± 2.1	1 087	0.95	0.90–1.00	0.071			
Perceived harmfulness of smoking							
Total	1 082			< 0.001			
Not at all	37 (3%)	1			1		
Neither harmless nor harmful	223 (21%)	1.37	0.64–2.96	0.421	1.43	0.59–3.41	0.427
Clearly harmful	822 (76%)	2.93	1.41–2.48	0.004	2.28	0.98–5.32	0.057
Quit attempts of use							
Total	1 068			< 0.001			
No attempts	561 (53%)	1			1		
One	317 (30%)	2.71	2.08–3.54	< 0.001	2.34	1.77–3.10	< 0.001
Two or more	190 (18%)	3.68	2.68–5.05	< 0.001	3.22	2.32–4.49	< 0.001
Perceived addictiveness of smoking							
Total	1 084			< 0.001			
Not at all	48 (4%)	1			1		
Neither nor	192 (18%)	1.02	0.55–1.91	0.941	0.99	0.50–1.95	0.971
Very addictive	844 (78%)	1.88	1.07–3.32	0.028	1.74	0.93–3.26	0.086
Advice to quit smoking							
Total	1084	1.83	1.34–2.61	< 0.001	1.65	1.17–2.32	0.004
Level of cigarette dependence							
Total	1 095			< 0.001			
No/low dependence	541 (49%)	1			1		
Moderate	261 (24%)	0.87	0.66–1.15	0.322	0.99	0.74–1.33	0.947
Strong	205 (19%)	0.51	0.37–0.70	< 0.001	0.63	0.44–0.89	0.008
Very strong	88 (8%)	0.35	0.22–0.56	< 0.001	0.43	0.26–0.71	0.001

Relative order of the outcome values used in the regression: not willing to quit; willing to quit after 6 months; willing to quit within 6 months.

OR^a^ Univariate analysis: information missing for 77/1095 cases.

OR^b^ Multivariate analysis: information missing for 51/1044 cases.

By multivariate analysis, earlier quit attempts within the past 6 months were strongly associated with the willingness to quit: one attempt to quit yielded an OR of 2.34 (95% CI 1.77–3.10) and two or more attempts 3.22 (95% CI 2.32–4.49). Having received advice to stop smoking increased the probability of smoking cessation by 65% (OR 1.65, 95% CI 1.17–2.32). The perception of the addictiveness of cigarette smoking showed a trend for willingness to quit among smokers (OR1.74, 95% CI 0.93–3.26, p = 0.08). The level of dependence was inversely associated with the willingness to stop smoking: with strong dependence the OR was 0.63 (95% CI 0.44–0.89) and with very strong dependence 0.43 (0.26–0.71). (Table 4).

## Discussion

We investigated factors affecting the willingness to quit snus use and cigarette smoking among young Finnish adult males. By multivariate analyses the willingness to quit snus usage was positively associated with the perception of snus-related health hazards and earlier quit attempts. The willingness to quit smoking was positively associated with earlier quit attempts and with getting advice to quit, and inversely associated with dependence.

Since there is no simple practical questionnaire available for evaluating snus dependence, we developed a preliminary two-question dependence test for snus users building on the two-question HSI for smokers. The level of dependence was similar for smokers and snus users: half of daily smokers and snus users reported low nicotine dependence and a quarter strong or very strong dependence.

Snus use was almost as common as smoking in this population (35% vs. 38%). Tobacco use within a population is often associated with educational level^32,33^, but our results did not show an association between snus use and educational background, unlike the situation for smokers. Our findings resemble the change in snus habits reported from Norway, where snus use has increased among young adults and it is equally common as smoking and not associated with educational background^34^. According to the Finnish School Health Promotion Studies, snus use is linked to socioeconomic factors, although not as strongly as smoking^1,5^. We have shown in a previous study that the total daily exposure time to snus is 2.5 h less among respondents with a secondary education than among respondents with basic education although the prevalence of snus use did not differ between the groups^2^. These findings may implicate that the readiness to use snus regularly is equally common, but socioeconomic factors related to higher education protects from extensive use.

Lately, an increase in snus use, although is still uncommon, has been reported among young Finnish females with a vocational education (prevalence 6%)^5,35^. In our study 7% of the female conscripts used snus and a quarter smoked daily or occasionally. These findings are interesting but cannot be generalised, since female conscripts are a selected group of respondents^36^. Also, the cohort of female conscripts in our study was very small. Nevertheless, females may turn out to be an important future target group for prevention of snus use and cessation efforts, if the snus epidemic continues to evolve in Finland as it has in Norway^37^.

Several studies show that most smokers want to quit^5,9,38^ and that knowledge about the negative health effects of smoking and receiving the advice to quit the habit increase the motivation to quit smoking^39,40^. We made the same observations (Table 4). Studies on the attitudes toward the health hazards of snus use and factors affecting the willingness to quit snus use in this age group have, on the other hand, apparently not been conducted previously.

In our study, approximately half of daily snus users and half of daily smokers wanted to quit using the tobacco product. This desire was not related to educational background. Almost 90% of daily smokers recognised smoking as being harmful to health, but only half of snus users considered snus as harmful. The willingness to quit snus was three-fold among the snus users who considered snus to be harmful compared to those who did not. The association between the perceived harmfulness of smoking and the willingness to quit was, on the other hand, weaker among smokers, but the perception that smoking is highly addictive did increase the willingness to quit. Smoking is indeed a well-known health hazard and this high level of awareness could explain this finding.

Interestingly, only 13% of snus users but 30% of smokers wanted to quit their habit during their military service. Earlier studies have shown that snus use is more common than smoking among physically active persons, particularly persons involved in team sports^41,42^. Our findings indicate that the knowledge about the adverse effects of snus on physical and psychological health is poorer than the effects of smoking. This circumstance might reduce the motivation to quit snus use and lower the threshold to start. The same overall lack of knowledge of the deleterious effects of snus might also explain the high interest in snus and the negligible impact of the educational background on reducing snus*.*

Snus exposes the user rapidly and for a prolonged time to high nicotine blood levels and this increases the risk of nicotine addiction when snus is used regularly^18,19^. The amount of nicotine in snus is not regulated^10^ nor is the nicotine content reported in standards terms on the snus containers, which makes it difficult to evaluate and compare the amount of nicotine exposure from the different available snus brands. Reliable statistical data on the strength of snus products used in the Nordic countries is available only from Norway, where strong snus (containing over 20 mg/g of nicotine) has rapidly gained market shares among young adults^34^. The trend seems similar in Finland, as indicated by custom reports on confiscated snus^43^. A small-scale survey (n = 273/660) was conducted in June 2020 in the Finnish Defence Forces as part of an ongoing snus prevention project: 61.5% of users used snus with a nicotine content of 21–30 mg/g and 24.5% with a nicotine content of 11–20 mg/g (Anelma Lammi, personal communication). These signals are worrying especially as the average daily duration of exposure to snus exceeded six hours among daily users^2^.

The six-question dependence test for snus users^26^ is impractical and it is rarely used in everyday practice in Finland. Instead, the two-question nicotine dependence test for snus users introduced in this study is quick and easy to use by healthcare professionals. The preliminary result from this short test is encouraging: the scale of dependence of snus was similar to the 2-questions HSI for smokers. Only very strong snus dependence was associated with the willingness to quit based on the univariate analysis. This association was lost in the multivariate analysis, as it was for the association between dependence and the perception that snus is addictive.

These findings implicate that snus habits and nicotine dependence differ somewhat from that of smokers. Many users may have a solid understanding of the addictiveness and harmfulness of smoking, but this may not be the case for snus. It is also possible that the preliminary two-question dependence test for snus users may not be predictive on the practical level, unlike HSI. Unfortunately, we were not able to compare the results with validated smokeless tobacco dependence tests^24,25^, because our questionnaire did not include all the required questions for this purpose. Therefore, evaluation of snus dependence with the two-question test needs further study and needs to be validated before being taken into routine use.

### Strengths and limitations

For this study, we recruited study subjects over a 3-year period (2014, 2015 and 2016) and reached a representative number of young Finnish males. The study was conducted in a military setting during the first week of service; this ensured that the responses reflected tobacco habits in general^8^. Our broad-spectrum questionnaire enabled analyses of nicotine addiction, attitudes towards tobacco and nicotine use, perceptions of health hazards, and perception of addictiveness of snus and cigarettes and provided basic prevalence information. To our knowledge, this is the first study to demonstrate predictive factors related to snus use. The results were statistically robust. The high response rate (72%) provides solid and representative data on the tobacco habits and attitudes towards tobacco use of males in this age group.

However, the cohort excludes approximately 25–30% of the males who chose non-military service or were exempted from military service because they were unsuitable due to circumstances like mental health problems or drug use. This may certainly have introduced some bias to the results. A major limitation of this study is that the preliminary two-question dependence test for snus users could not be compared with a validated snus dependence test or to cotinine measurements. We attempted to introduce a shorter assessment model that could accurately assess dependence, but due to limited resources, we were unable to provide validation to support the item selection. Thus, direct comparison between the two-item measure and serum cotinine levels must be documented before the abbreviated measure can serve as a proxy for dependence.

## Conclusions

Smoking and snus use is common among young Finnish adult males. The degree of willingness to quit snus use is associated with the perception of health hazards, and with previous quit attempts. In general snus use is considered less harmful and less addictive than smoking. A history of previous quit attempts and of getting advice to stop smoking increase the willingness to quit smoking.

Our findings support the significance of early health promotion efforts and the need for spreading information on the negative health effects of snus. Easily applicable methods to estimate and recognise nicotine addiction are needed to support quitting snus use. Our two-question snus dependence test could be helpful, but it needs to be validated by a direct comparison to serum cotinine levels.

## Data Availability

The datasets generated and/or analysed during the current study are not publicly available due to unpublished material that will be used in future publications but is available from the corresponding author upon reasonable request.

## Abbreviations

CI: Confidence index
HSI: Heaviness of Smoking Index
OR: Odds ratios
WHO: World Health Organization
